# Innovation Strategy of Cultivating Innovative Enterprise Talents for Young Entrepreneurs Under Higher Education

**DOI:** 10.3389/fpsyg.2021.693576

**Published:** 2021-08-23

**Authors:** Xiao Yu, Baoge Zhang

**Affiliations:** College of Teacher Education, Ningbo University, Ningbo, China

**Keywords:** innovative talents cultivation, time series analysis, higher education, mental health level, young entrepreneurs

## Abstract

A time series model is designed based on the backpropagation neural network to further optimize the innovation and development of new ventures. The specific situation of two factors is primarily analyzed as follows: the supply and demand ratio of enterprise talents and the state of entrepreneurship in the development of new ventures. The results show that the potential demand of future enterprises for big data talents can be obtained by fitting prediction sequences. Based on the Backpropagation–Autoregressive Integrated Moving Average model, the post modeling and prediction are carried out, and the coefficient 0.6235 is obtained by substituting the equation of Pearson's correlation coefficient. The analysis results suggest that the matching needs to be strengthened between the cultivation of innovative talents in universities and the demand trend of big data-related positions in enterprises. Moreover, there is a mismatch between the cultivation of innovative talents and the demand for innovative talents. Meanwhile, the mental health level of young entrepreneurs is concerned. The mental health status of young entrepreneurs is compared with the national norm data through the questionnaire survey and statistical data analysis. The results reveal that the mental health level of young entrepreneurs is significantly lower than that of the national norm, and the proportion of anxiety and depression is 29.4% and 27.5%, respectively. Considering the professional characteristics and competitive environment of young entrepreneurs, busy work and the multiple missions given by society to entrepreneurs are the major reasons for their pressure, and mental health problems are serious.

## Introduction

The arrival of a knowledge-based society has promoted the reform of economic development mode, and the driving force led by innovation has become the key to a new round of international competition. Innovation is related to the development of national strategy. The driving force to enhance the national innovation ability lies in deepening the reform of innovation and entrepreneurship education in universities and cultivating innovative and entrepreneurial talents (Lu and Xue, 2017). The cultivation of innovation and entrepreneurship education talents aims to cultivate innovative entrepreneurs through the innovation of entrepreneurship education and injects innovative vitality into the country, economy, employment, and other social development. Innovation and entrepreneurship is the key to success in the international competition of knowledge society (Zhou and Tian, 2019). Developing innovation and entrepreneurship education and cultivating innovative enterprise talents are not only the developmental trend of higher education in the world but also the internal generating force for implementing the innovation-driven strategy, building an innovative country, and developing an entrepreneurial economy in China. Entrepreneurial economy is not only the internal requirement of optimization and adjustment of industrial structure but also the main driving factor to promote the economic growth (Xi et al., 2020), which is essentially a kind of entrepreneur economy. Entrepreneurship and entrepreneurial activities are its core production factors, and innovation is its norm. Especially, small and medium-sized enterprises (SMEs), as the source power, play an important role in the entrepreneurial economy. SMEs account for a high proportion of the current economic development of all countries in the world and are known as “machines for creating jobs.” The new strength of entrepreneurs of SMEs comes from young entrepreneurs, who receive systematic entrepreneurship education in higher education to help start-ups (Jin and Liao, 2017). Wu and Wu (2017, 2019) showed that with the acceleration of social development, the research on entrepreneurship was gradually increasing, and there were booming studies on innovation and entrepreneurship. Wu et al. (2019) fully illustrated the importance of innovation and entrepreneurship education and entrepreneurship to social and economic development. Besides, Wu et al. (2018) also fully explained the significance of innovation and entrepreneurship courses for college students.

At present, China is popularizing higher education. The continuous growth of the number of college graduates and the shortage of jobs have brought huge social employment pressure. As a whole, labor demand is still more than supply in the employment market, showing a tense situation. Innovation and entrepreneurship education aims to change the passive concept of employment of students into active, create new jobs, close the relationship between enterprises and universities, and promote the transformation of scientific and technological achievements (Ding, 2017). Therefore, how to cultivate top-notch innovative talents to adapt to the new era has become a major issue in the process of education construction. With the acceleration of a new round of scientific and technological revolution and industrial change, the whole society has higher requirements for talents, especially top-notch innovative talents (Linton and Klinton, 2019). In order to meet the new needs of social development in the new era for top-notch innovative talents, the innovation ability and practical ability of college students need to be further strengthened. However, compared with enterprises, research universities lag behind in frontier knowledge acquisition and new technology application; meanwhile, knowledge transfer itself does not mean that innovation and practical ability of students will be generated (Kickul et al., 2018; Hasan et al., 2019). Therefore, to overcome the limitations of universities in talent cultivation, it is more urgent and necessary for enterprises to participate in cultivating top-notch innovative talents (Huang, 2020). Wu et al. also further explained that employee innovation was of great significance for enterprises, and employee innovation performance could effectively promote the healthy development of enterprises. However, in China, for a long time, universities are the main or even the only subject of talent cultivation, while enterprises are market-oriented economic entities. Most enterprises are still on the demand side for talents, so it is difficult to bring talent cultivation into their career fields (Weichen, 2020). Therefore, if the main role of talent cultivation of enterprises is effectively played to promote the transformation of enterprises from talent demand to talent supply and demand, it will help to promote the all-round integration of talent cultivation supply and industry demand in structure, quality, and level, thereby improving the quality of cultivating top-notch innovative talent (Chen, 2019).

Based on the above background, a comparative study is made on the cultivation scheme of innovative talents in universities and the demand characteristics of enterprises for innovative talents, and the cultivation of innovative talents in higher education is explored from the perspective of talent demand. Meanwhile, the mental health of young entrepreneurs is concerned, to provide a reference for the reform of cultivation mode of innovative talents in universities. The algorithm related to artificial intelligence is applied to the cultivation of innovation and entrepreneurship talents in colleges, which has strong innovation in methods. Besides, this exploration has significant application value and practical significance, which is suitable for the needs of the era of big data at this stage (Kong and Zhao, 2020; Deng et al., 2021).

Under the above background, the enterprise demand and talent model are designed based on the related concepts of backpropagation neural network (BPNN). Meanwhile, the research on the mental health of entrepreneurs aims to analyze the specific status of factors closely related to the development of enterprises and promote the healthy, stable, and orderly development of enterprises (Li and Cao, 2020; Yin et al., 2020).

## Methods

### The Matching Problem Between Enterprise Talent Demand and College Talent Cultivation

The new industry brings new information age and changes the demand for talents in this field. It is a major test for contemporary educators to meet the needs of new high-quality information technology talents in the new normal society under “innovation-driven” and promote the deep integration of education and enterprises (Beiping et al., 2019). As the main institutions of high-end talent education and cultivation, universities need to integrate resources as soon as possible, adjust the specialty setting and structure as needed, and reform the cultivation scheme, thus meeting the requirements of the society in the era of big data for cultivating new big data talents. Enterprises have higher and higher requirements for all aspects of the quality of the unemployed. The real-time position data of the recruitment platform, as the embodiment of the demand of enterprise for job seekers, can provide data support for establishing a college cultivation policy.

According to the existing data, a certain number of scholars in China and foreign countries have done relevant studies on the matching degree between specialty setting and work of graduates. Pratt et al. discussed that the employment direction of graduates did not match their major (Pratt et al., 2019); Barry conducted a career evaluation questionnaire on college graduates, multiplied the evaluation results of each dimension of the job seeker with the weight of the evaluation dimension to get the final score, and finally judged the fit degree between the job seeker and the post through the score (Barry, 2021); Wehman et al. (2018) investigated the employment matching degree of college students in recent 5 years from multiple dimensions through the questionnaire of the job matching degree of the major of the college graduates and initial employment; Assari (2019) obtained the contradiction between the talent cultivation setting and the employment matching degree theoretically and put forward some suggestions for the problem of college major setting. Throughout the research resources of talent cultivation in universities and employment matching degrees, the traditional way of questionnaire or theoretical way of industrial structure analysis are mostly used, while there are still few research methods of modeling and quantifying post data based on data mining. However, the existing resources in China and foreign countries show that data quantification contributes to analyzing employment status and trend research.

With the popularity of the Internet, more and more enterprises release their recruitment information through the recruitment network platform. The emergence of online recruitment can effectively solve the limitations of existing data statistics, which is also the information channel that can best reflect the market demand for talents; recruitment information is concentrated on the Internet, which is characterized by a large amount of data, so it is difficult to accurately obtain effective intelligence only by the search engine (Shuzhen, 2019). In view of the above problems, based on the improved time series method suitable for the era of big data, the demand trend of big data jobs is predicted by integrating the data of the online recruitment platform. Moreover, based on the developmental trend of the new information society, from the perspective of the combination of data and theory, the major matching degree of talents in universities is studied. It aims to accurately understand the characteristics of market demand and provide countermeasures and suggestions for talent cultivation and curriculum setting in universities.

### Time Series Model for Enterprise Talent Demand

The talent demand series of enterprises is greatly influenced by time and has certain regularity. The time series model method or the corresponding combination model is often used for model construction and prediction analysis (Salgotra et al., 2020). Time series refers to that with certain quantity, the same statistical index, and arranged in time developmental order; the time series analysis can quantitatively reveal the development and change of a phenomenon through the dynamic data processing method of random process and mathematical statistics, providing the support for users to solve practical problems; the core of time series analysis method prediction lies in mining a certain rule from the changing data over time and using it to estimate the data of a certain time in the future (Picoli et al., 2018). Time series model prediction is easy to use and has high short-term prediction accuracy, which can use historical data to predict the future. The time series model whose mean and variance do not change significantly in a certain period of time is called the stationary time series model. Stationary time series strictly eliminate periodic changes and usually achieve prediction through establishing a linear model (Yang and Liu, 2019).

Simple stationary time series mainly include *AR*(*p*), *MA*(*q*), and *ARMA*(*p, q*) models, and nonstationary time series include Autoregressive Integrated Moving Average (*ARIMA*) models.

In practice, the time series obtained are often nonstationary, so the *ARIMA* model is more commonly used to solve the practical problems of nonstationary series. In essence, it increases the difference times based on the *ARMA* model (Schultzberg and Muthén, 2018). The commonly used difference is calculated as follows (1):

(1) $$\begin{array}{l} \phi \left(B\right)\Delta^{d}x_{t}=\delta +\Theta \left(B\right)u_{t} \end{array}$$

where δ is the residual and *u* is the mean of time series.

The difference operator is as follows:

(2) $$\begin{array}{l} \Delta x_{t}=x_{t}-x_{t-1}=\left(1-B\right)x_{t} \end{array}$$

(3) $$\begin{array}{l} \Delta^{2}x_{t}=x_{t}-x_{t-1}=\left(1-B\right)x_{t-1}={\left(1-B\right)}^{2}x_{t} \end{array}$$

(4) $$\begin{array}{l} \Delta^{d}x_{t}={\left(1-B\right)}^{d}x_{t} \end{array}$$

Set:

(5) $$\begin{array}{l} w_{t}=\Delta^{d}x_{t}={\left(1-B\right)}^{d}x_{t} \end{array}$$

The value *W*_*t*_ at time *t* is calculated as follows (6):

(6) $$\begin{array}{l} W_{t}=\phi_{1}W_{t-1}+\phi_{2}W_{t-2}+\ldots \\ \;+\phi_{p}W_{t-p}+\delta +u_{t}+\theta_{1}u_{t-1}+\theta_{2}u_{t-2}+\ldots +\theta_{q}u_{t-q} \end{array}$$

*p* is the autoregressive order and *q* is the moving average order.

Auto-correlation function (ACF) and part auto-correlation function (PACF) are used to identify the order of the model. *ACF* describes the linear correlation between the current observation value and its past time value. The autocorrelation coefficient *p*(*k*) is expressed as follows (7):

(7) $$\begin{array}{l} p\left(k\right)=\frac{r\left(k\right)}{\sigma^{2}} \end{array}$$

where *r*(*k*) is the variance and *k* is the delay of time series.

In order to eliminate the influence of *p*(*k*) doping other variables, *PACF* is used to judge and describe the linear correlation of the sequence observations under the condition of eliminating the interference of intermediate observations, defined as follows (8):

(8) $$\begin{array}{l} \rho_{X_{t},X_{t-k}|X_{t-1},\ldots ,X_{t-k+1}}=\frac{E\left[\left(X_{t}-ÊX_{t}\right)\left(X_{t-k}-ÊX_{t-k}\right)\right]}{E\left[{\left(X_{t-k}-ÊX_{t-k}\right)}^{2}\right]} \end{array}$$

If the time series {*Y*_*t*_, *t* = 1, 2, …} is periodic, it is necessary to make a periodic difference for {*Y*_*t*_}. The specific steps are as follows:

(9) $$\begin{array}{l} \nabla_{s}Y_{t}=Y_{t}-Y_{t-s}\left(t>s\right) \end{array}$$

(10) $$\begin{array}{l} {\nabla^{2}}_{s}Y_{t}=\left(Y_{t}-Y_{t-s}\right)-\left(Y_{t-s}-Y_{t-2s}\right)\left(t>2s\right) \end{array}$$

The stationary sequence {*X*_*t*_} is obtained by *D* times the periodic difference of {*Y*_*t*_}.

(11) $$\begin{array}{l} \left\{X_{t}\right\}=\nabla_{s}^{D}Y_{t}\left(t>D\right) \end{array}$$

Then, the model expression changes to

(12) $$\begin{array}{l} \phi \left(B\right){\left(1-B^{s}\right)}^{D}Y_{t}=\Theta \left(B\right)\varepsilon_{t} \end{array}$$

Among them,

(13) $$\begin{array}{l} \phi \left(B\right)=1-\phi_{1}B^{s}-\phi_{2}B^{2}s-\ldots -\phi_{p}B^{ps} \end{array}$$

(14) $$\begin{array}{l} \Theta_{Q}\left(B\right)=1-\Theta_{1}B^{s}-\Theta_{2}B^{s}s-\ldots -\Theta_{p}B^{ps} \end{array}$$

The model is generally expressed as follows (15):

(15) $$\begin{array}{l} \varphi \left(B\right)\phi \left(B\right){\left(1-B\right)}^{d}{\left(1-B^{s}\right)}^{D}Y_{t}=\theta \left(B\right)\Theta_{Q}\left(B\right)\varepsilon_{t} \end{array}$$

where φ(*B*) is the autoregressive characteristic polynomial of {*Y*_*t*_}, Θ(*B*) is the seasonal moving average characteristic polynomial, ε_*t*_ is the residual, *B* is the delay operator, and *p* is the order of seasonal regression.

### Combination of BPNN and Time Series Model

**Figure 1:**
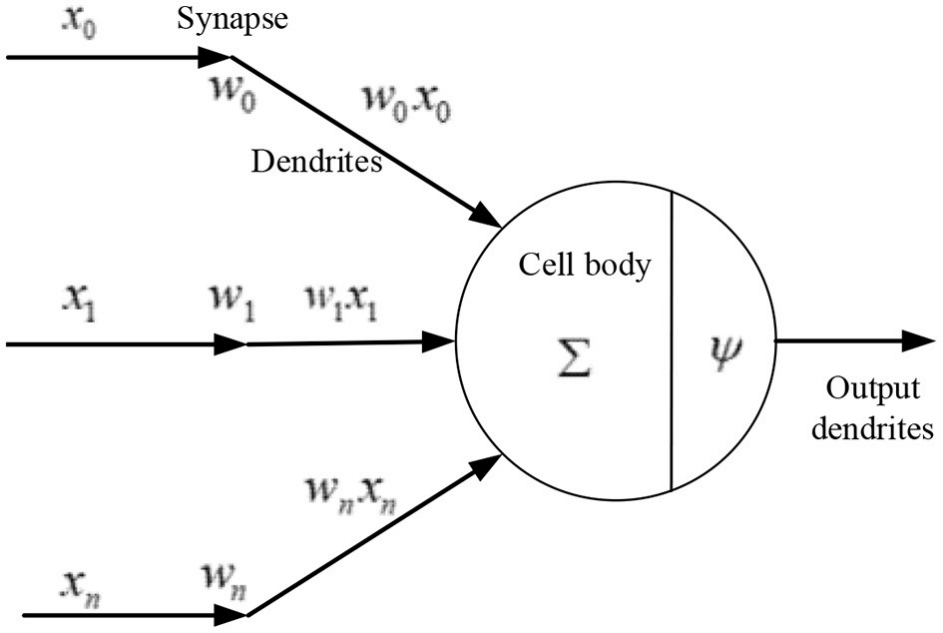
Structure of a simple neuron.

The neural network is a data processing model based on the enlightenment of the human neural system. A single neuron is an essential element of the artificial neural network, which is designed according to biological brain neurons. The simplest neuron is composed of multiple inputs and one output (Ruan et al., 2020), as shown in Figure 1. Neural networks adjust their own structure according to the external information and process the original data by using the weights between neural networks because they can make nonlinear processing and parallel computing (Li et al., 2020).

**Figure 2:**
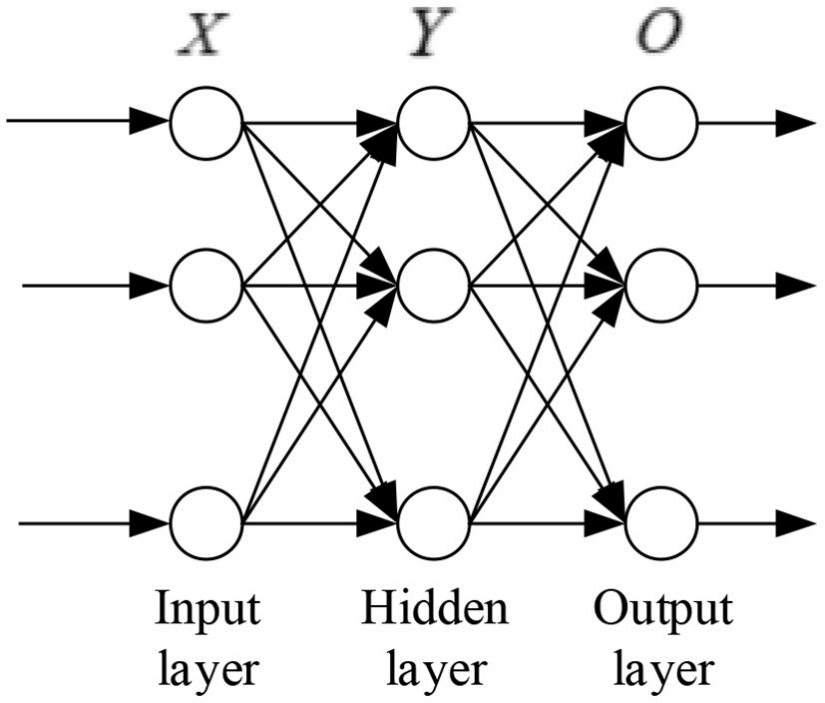
Structure diagram of back propagation neural network.

Backpropagation neural network is a one-way neural network with three layers, namely, input layer, hidden layer, and output layer. BPNN is widely used in data processing, face recognition, fitting function, and prediction (Zhang et al., 2020). BPNN minimizes the error between the predicted value and real value. In general, the error revision of the neural network is carried out by learning and modifying the weights and thresholds of neurons until the iteration is stopped or the error reaches the preset goal (Li et al., 2020). The basic structure of BPNN mainly includes the following parts (Figure 2).

Input vector is

(16) $$\begin{array}{l} X={\left(x_{1},x_{2},\ldots ,x_{i},\ldots ,x_{n}\right)}^{T} \end{array}$$

Output vector is

(17) $$\begin{array}{l} O={\left(o_{1},o_{2},\ldots ,o_{k},\ldots ,o_{l}\right)}^{T} \end{array}$$

The output vector of the hidden layer is

(18) $$\begin{array}{l} Y={\left({\text{y}}_{1},{\text{y}}_{2},\ldots ,{\text{y}}_{i},\ldots ,{\text{y}}_{m}\right)}^{T} \end{array}$$

The connection weight matrix from the input layer to the hidden layer is

(19) $$\begin{array}{l} V=\left(V_{1},V_{2},\ldots ,V_{i},\ldots ,V_{m}\right) \end{array}$$

The connection weight matrix from the hidden layer to the output layer is

(20) $$\begin{array}{l} W=\left({\text{W}}_{1},{\text{W}}_{2},\ldots ,{\text{W}}_{k},\ldots ,{\text{W}}_{l}\right) \end{array}$$

### Structure of BP–ARIMA Model

The basic principles of the combination model are to combine a few different single prediction models or some information provided by several single factors in a certain way to get a more integrated and credible model. It will make the single model complementary to each other and improve the prediction accuracy of the model, making the prediction result more ideal (Liu et al., 2020).

A new combination prediction model is established, which contains the advantages of multiple single prediction models. Some scholars have proposed ARIMA, neural network combination model, in recruitment demand data prediction and achieved good prediction results. The ARIMA model can make up for seasonal errors and is suitable for the research of recruitment data. However, the BP–ARIMA model has not been used in the research at present. The research is feasible and advanced.

A three-layer BPNN cultivation includes the following four parts.

(1) Network initialization, sample X input, and desired Y output.

For the sample input sequence and the sample expected value output sequence, the network input layer endpoint is set as *n*, and the output layer endpoint is set as *m*. The number of hidden layer nodes *l* is calculated as follows (21):

(21) $$\begin{array}{l} l=\sqrt{m+n}+d \end{array}$$

In equation (21), 1 ≤ *d* ≤ 10.

(2) Calculation of the hidden layer and the output layer. The weight of each layer is connected with the threshold, and the hidden layer is calculated by the input signal of the input layer.

(22) $$\begin{array}{l} H_{j}=f\left(\overset{n}{\underset{i=1}{\sum }}w_{ij}x_{i}-a_{i}\right) \end{array}$$

The output layer is calculated as follows (23):

(23) $$\begin{array}{l} O_{k}=\overset{n}{\underset{j=1}{\sum }}H_{j}w_{jk}-b_{k} \end{array}$$

(3) Error calculation and adjustment of weight and threshold. The initial weight and threshold are adjusted by the error value.

(24) $$e_{k}=\frac{1}{2}\sum_{k=1}^{m}(Y_{k}-O_{k}{)}^{2}$$

(4) The global error is obtained by forward propagation and backpropagation.

(25) $$\begin{array}{l} E=\frac{1}{2t}\overset{m}{\underset{t=1}{\sum }}\overset{m}{\underset{k=1}{\sum }}{\left(Y_{k}-O_{k}\right)}^{2} \end{array}$$

### Data Acquisition of Enterprise Innovative Talent Based on the Improved Model

Enterprise recruitment demand is affected by many factors. To explore the status quo of the matching between big data job demand of enterprise and college talent cultivation, first, python is used to obtain and preprocess the data of enterprise job demand, and the improved BP–ARIMA model suitable for this scenario is used for modeling; it provides data support for the investigation of demand status and trend of enterprises.

Most of the existing research data come from the web pages of major online recruitment platforms, which contain the requirements of enterprises for job seekers in all aspects of skills. However, for the recruitment information that existed on the web pages, most of the requirements for job seekers are semi-structured or unstructured text information. Based on python language, the job information in the recruitment website is crawled, and the data are obtained, including the job name, recruitment company name, salary, company address, education requirements, and experience requirements of all kinds of current positions on the website. After crawling, the original data may be incomplete or abnormal, so it is necessary to preprocess the initial data. The data of the website post demand table obtained are relatively regular, and it only needs to clean up and sort out the data.

For the time series that need to build a time series model, the actual input series are often nonstationary time series. If the time series analysis is directly used for modeling and fitting, it will easily lead to a pseudo regression phenomenon. At this time, it is necessary to transform the nonstationary time series into stationary time series according to the scatter plot, data transformation, and difference operation of time series; in practice, the truncation or tailing of autocorrelation graph and partial autocorrelation graph are often used to determine the stability of the sequence, and the difference operation is used to process the sequence. The augmented Dickey–Fuller test method is used to test the stationarity of time series. It is assumed that time series have unit roots. If the sequence is a stationary time series, the test results show that the statistics are significantly <1, 5, and 10% confidence critical values, which will be very close to 0. If the above conditions are not satisfied, the sequence is judged to be nonstationary time series. After testing, the time series model data met the requirements of the stationarity test.

### Construction of Prediction Model for Enterprise Innovative Talent Demand

After the difference times are determined, it is necessary to determine the order of the AR model and MA model in the ARIMA model, as well as the value of seasonal parameters. At this time, it is necessary to identify the model and estimate the parameters. Then, ARIMA model checking can be carried out (Qiu et al., 2021). The sequence formed by the time series autocorrelation function of *x*_*t*_ lagging 1 period is represented as *x*_*t*−1_, and the sequence lagging 2 periods is represented as *x*_*t*−2_. The interdependence degree *r*_*k*_ between two data points with phase *k* difference in the sequence is expressed as follows (26):

(26) $$\begin{array}{l} r_{k}=\frac{\sum_{t=k+1}^{n}\left(x_{t}-\bar{x}\right)\left(x_{t-k}-\bar{x}\right)}{\sum_{t=1}^{n}{\left(x_{t}-\bar{x}\right)}^{2}} \end{array}$$

The sequence average $\bar{x}$ is calculated as follows (27):

(27) $$\begin{array}{l} \bar{x}=\frac{1}{n}\sum_{t=1}^{n}x_{t} \end{array}$$

Then,

(28) $$\begin{array}{l} \phi_{k,j}=\phi_{k-1,j}-\phi_{kk}\phi_{k-1,k-j} \end{array}$$

(29) $$\begin{array}{l} \phi_{kk}=\frac{\rho_{k}-\sum_{j=1}^{k-1}\phi_{k-1,j}\rho_{k-j}}{1-\sum_{j=1}^{k-1}\phi_{k-1,j}\rho_{j}} \end{array}$$

(30) $$\begin{array}{l} \phi_{11}=\rho_{1} \end{array}$$

For the seasonal model with a period of 12, the trend components are decomposed into trend, seasonal, and residuals. The model is defined as follows:

(31) $$\begin{array}{l} z_{t}=T_{t}+S_{t}+\varepsilon_{t} \end{array}$$

where *T*_*t*_ is the trend feature of the series, *S*_*t*_ is the seasonal feature of the series, and ε_*t*_ is the steady-state noise feature of the series.

(32) $$\begin{array}{l} T_{t}=T_{t-1}+\beta_{t-1}+\eta_{t} \end{array}$$

(33) $$\begin{array}{l} \beta_{t}=\beta_{t-1}+\xi_{t} \end{array}$$

where η_*t*_ represents the white noise process with variance $\sigma_{\eta }^{2}$ and mean value 0; ξ_*t*_ indicates the white noise process with variance $\sigma_{\xi }^{2}$ and mean value 0; ε_*t*_ stands for the white noise process with variance $\sigma_{\varepsilon }^{2}$ and mean value 0; β is a constant.

The above model can be simplified as follows:

(34) $$\begin{array}{l} \left(1-B\right)T_{t}=\beta +\eta_{t} \end{array}$$

(35) $$(1+B+\ldots \text{+}B^{\text{11}}\text{)}S_{t}=\omega_{t}$$

Then, the difference sequence is expressed as follows:

(36) $$\begin{array}{l} \omega_{t}=\left(1-B\right)\left(1-B^{12}\right)z_{t}=\left(1-B^{12}\right)\eta_{t}+{\left(1-B\right)}^{2}\omega_{t}+\left(1-B\right)\\ \;\left(1-B^{12}\right)\varepsilon_{t} \end{array}$$

In this case, the auto-covariance of the sequence is expressed as follows:

(37) $$\begin{array}{l} \rho_{1}=-\frac{\sigma_{\varepsilon }^{2}+2\sigma_{\omega }^{2}}{2\sigma_{\varepsilon }^{2}+\sigma_{\eta }^{2}+3\sigma_{\omega }^{2}} \end{array}$$

(38) $$\begin{array}{l} \rho_{12}=\frac{2\sigma_{\varepsilon }^{2}+\sigma_{\eta }^{2}}{2(2\sigma_{\varepsilon }^{2}+\sigma_{\eta }^{2}+3\sigma_{\omega }^{2}\text{)}} \end{array}$$

(39) $$\begin{array}{l} \rho_{11}=\rho_{13}\text{=}\frac{\sigma_{\varepsilon }^{2}}{2(2\sigma_{\varepsilon }^{2}+\sigma_{\eta }^{2}+3\sigma_{\omega }^{2}\text{)}} \end{array}$$

Among them, ρ_1_, ρ_11_, ρ_12_, and ρ_13_ are autocorrelation coefficients.

Therefore, the operation of separating the seasonal trend can be performed on the time series, and the function addition model is used to separate the time series data into the trend, seasonal, and residuals.

For the time series model, the more the cultivation data are the higher the accuracy of the model is. For the model with few parameters, BIC measurement is generally chosen. BIC measurement criterion is the Bayesian information criterion, which uses subjective probability to estimate some unknown states in the model. Using the Bayesian equation, the probability of occurrence is transformed into a posteriori probability and modified. Finally, the optimal decision can be made according to the expected value and modified probability (Yu et al., 2021).

For the maximum model *M*_*i*_ with the given data, the posterior probability is defined as follows:

(40) $$\begin{array}{l} P\left(M_{i}|{\text{y}}_{1},\ldots ,y_{n}\right)=\frac{P\left({\text{y}}_{1},\ldots ,y_{n}|M_{i}\right)P\left(M_{i}\right)}{P\left({\text{y}}_{1},\ldots ,y_{n}\right)} \end{array}$$

where *P*(*y*_1_, …, *y*_*n*_|*M*_*i*_) is the edge probability of the model and *P*(*M*_*i*_) is the fixed value. Then, the maximum posterior probability is equivalent to the edge probability of the maximum model.

(41) $$\begin{array}{l} P\left({\text{y}}_{1},\ldots ,y_{n}|M_{i}\right)=\int_{\theta_{i}}L\left(\theta_{i}|{\text{y}}_{1},\ldots ,y_{n}\right)\delta_{i}\left(\theta_{i}\right)d\theta_{i} \end{array}$$

θ_*i*_ is the parameter vector, δ_*i*_(θ_*i*_) is the probability distribution, and *L* is the likelihood function.

There are three ways to predict the model as follows: difference equation method, summation method, and weighted average method. The prediction of the seasonal model is directly calculated by the difference equation method, which is simple and easy to operate. Therefore, the difference equation is directly expressed as follows:

(42) $$\begin{array}{l} z_{t+1}=\varphi_{1}z_{t+1-1}+\varphi_{1}z_{t+1-12}+\varphi_{1}z_{t+1-13}+\alpha_{t+l}-\theta \alpha_{t+l-1}\\ \;-\Theta \alpha_{t+l-12}+\Theta \theta \alpha_{t+l-13} \end{array}$$

On the premise of data fitting, the model with parameter estimation is used to predict the future situation of the series.

The above prediction methods are robust to moderate changes of parameter values. With the fitting and iterative prediction of the predicted value and the real value of the data many times, the accuracy will be higher with the progress of the prediction. Python is used to build neural network modules. Based on the network cultivation time cost, network hardware application cost, and other factors, after repeated testing and cross-evaluation of the error, the first-to-fourth order difference data of the residual sequence are taken as the input variable, and the residual sequence is taken as the output variable. The hidden node is set to 8 and the output node is set to 1.

### Enterprise Talent Demand and College Cultivation Mode

The influencing factors of education development are complex, which not only rely on the internal factors of education policy but also are affected by the external factors of the education development environment. How to cultivate a group of elite talents that enterprises really need is the primary task of education development. The talent cultivation policy of universities is closely related to the recruitment needs of enterprises and the local education development environment. The current situation and trend of market demand for talents are obtained through the combination model of “time series + neural network,” and it is taken as the internal factor of education development with the trend matching research of big data specialty setting in universities. The newly increased situation of big data major in each province is regarded as the external factor of the education environment. With the two evaluation results as the reference factors of educational resources allocation reform in Jiangsu Province, countermeasures and suggestions are put forward for promoting the optimization and sustainable development of educational resources (Feng and Chen, 2020).

**Figure 3:**
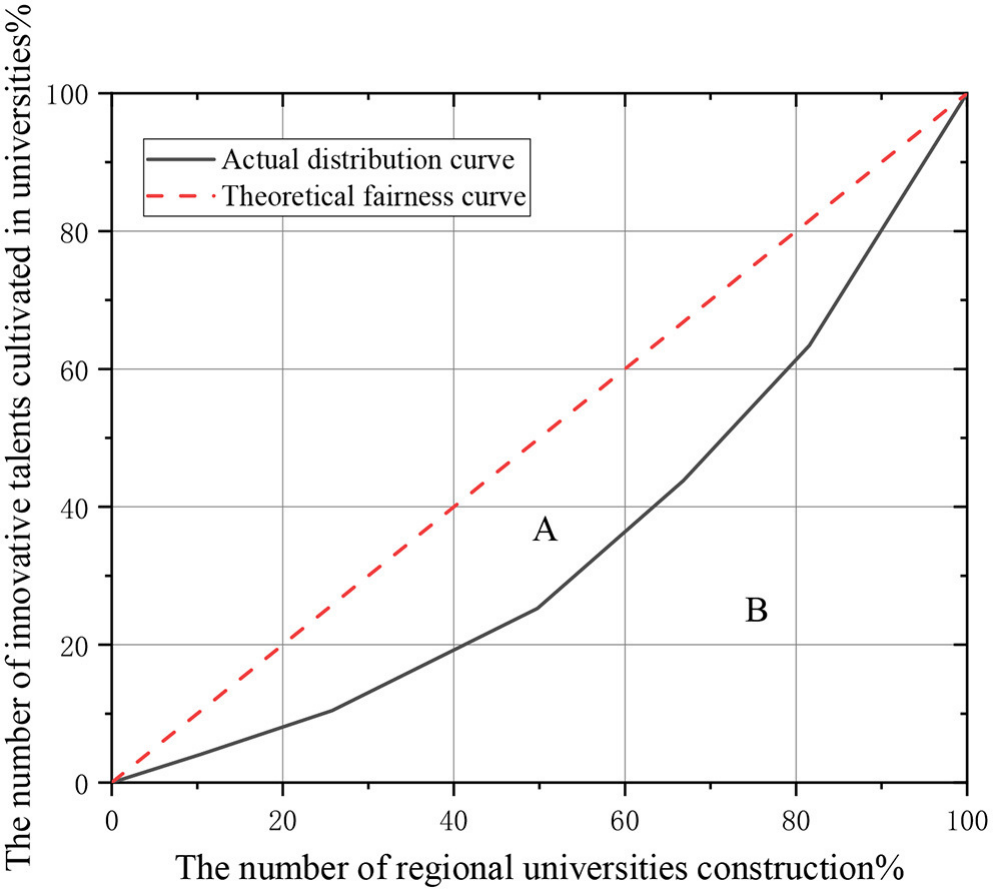
Lorenz curve analysis of educational resources and talent cultivation in universities.

Research is conducted based on the Lorenz curve, and the Gini coefficient is calculated to quantitatively describe the matching degree between the newly increased situation of big data major in each province and the education level of local universities. *x* represents the cumulative percentage of the number of universities in the region, and *y* denotes the new incremental percentage of the corresponding big data major. B stands for the area under the actual distribution curve, and A means the difference between the area under the theoretical fairness curve and the area under B. Figure 3 shows a Lorentz curve of specialty setting and education level of regional universities.

If the number of groups is *n* and the number of innovative talents cultivation majors in each group is *Y*_*i*_, the area calculation equation *P* of each group is as follows:

(43) $$\begin{array}{l} S_{p}=\frac{1}{2}AB\left(AC+BD\right) \end{array}$$

When the number of groups is *n*,

(44) $$\begin{array}{l} y_{i}=\frac{Y_{i}}{\overset{n}{\underset{i=1}{\sum }}Y_{i}} \end{array}$$

Then,

(45) $$\begin{array}{l} S_{B}=\overset{n}{\underset{i=1}{\sum }}\frac{1}{2n}\frac{\overset{n-1}{\underset{i=1}{\sum }}Y_{i}+\overset{n}{\underset{i=1}{\sum }}Y_{i}}{\overset{n}{\underset{i=1}{\sum }}Y_{i}} \end{array}$$

(46) $$\begin{array}{l} S_{A}=\frac{1}{2n}-\frac{n\overset{n-1}{\underset{i=1}{\sum }}Y_{i}-\left(\overset{n-1}{\underset{i=1}{\sum }}Y_{i}+\overset{n}{\underset{i=1}{\sum }}Y_{i}\right)}{\overset{n}{\underset{i=1}{\sum }}Y_{i}}\\ \;=\frac{1}{2n}\left(2n-2\sum \sum y_{i}+2\sum y_{i}\right)-\frac{n+1}{2n} \end{array}$$

(47) $$G=\frac{S_{A}}{S_{A}+S_{B}}=1-2\underset{k\to \infty }{\lim}\sum \frac{1}{2n}\frac{\overset{i-1}{\sum Y_{i}}+\overset{i}{\sum Y_{i}}}{\overset{n}{\sum Y_{i}}}$$

Finally, (48) can be obtained as follows:

(48) $$\begin{array}{l} G=2S_{A}=\frac{2}{n}\left(y_{1}+2y_{2}+\ldots +ny_{n}\right)-\frac{n+1}{n} \end{array}$$

The matching coefficient *G* is valued between 0 and 1. The closer the coefficient is to 1 the worse the matching degree is, that is, the higher the matching degree between the regional professional allocation and its education level is; on the contrary, the closer the coefficient is to 0, the better the matching degree is.

Pearson's correlation coefficient is used to study the correlation matching degree between the number of innovative talents in universities and the trend of demand of enterprises for innovative talents. The number of innovative talents cultivation and the trend of demand in the corresponding years of enterprises are taken as variable indexes; Pearson's correlation coefficient requires that the variable is continuous, and the year is taken as the time variable of the variable index, which can be regarded as a continuous variable; the change of the number of specialty settings in universities is determined by the ministry of education and the relevant national policies every year, while the demand for talents in enterprises comes from the market demand so that the variables are independent of each other, which conforms to the constraints of the model. Considering that universities need to train talents for 4 years, the number of innovative talents in 2015 is evaluated according to the demand of enterprises in 2020. The corresponding relationship of other year variables is the same. The number of innovative talents cultivation in universities from 2015 to 2020 corresponds to the number of recruitment predicted by the demand of enterprises from 2020 to 2025.

### Design and Reliability and Validity Test of Entrepreneur Mental Scale

The demand for enterprise talents will exert a certain impact on the enterprise, and the mental health of entrepreneurs is also a crucial factor affecting the development of enterprises. As the social rhythm is accelerated, young entrepreneurs are facing intense work and life pressure and various stressful events, and they are one of the highly stressed groups. Due to the particularity of the nature of the work, entrepreneurs are the group with a high incidence of psychological diseases. To evaluate the mental health of young entrepreneurs, the self-rating scale is mainly used to measure the level of individual mental health. Symptom checklist 90 (SCL-90) is the most commonly used tool in the mental health survey of Chinese entrepreneurs. Xia et al. (2021) analyzed the entrepreneurship of 6,108 employees and 2,075 entrepreneurs from 29 cities in different provinces of China. With this study as a reference standard, the differences between the study area and national average levels are compared. Through empirical research, young entrepreneurs in Shanghai are taken as the research object, including Internet and e-commerce, financial industry, education and cultivation, computer communication, and other industries which are emerging and developing rapidly, with significant characteristics of the times and high public awareness. Totally, 168 private enterprises are involved, and the selected respondents are mainly the top managers of various enterprises. SCL-90 developed by Derogatis is widely used in clinical and scientific research of mental health. The scale has 10 dimensions as follows: somatization, depression, hostility, terror, paranoia, anxiety, obsessive-compulsive symptoms, interpersonal sensitivity, mental illness, and others. The subjects are measured with SCL-90. There are 90 items, with a five-point scale from 1 “asymptomatic” to 5 “severe.” The homogeneity reliability of SCL-90 is 0.97, and that of each subscale is more than 0.69; the test–retest reliability is more than 0.7, and the content validity and structure validity are also good. In the form of a paper questionnaire, a total of 180 questionnaires are distributed and 165 are recovered. After screening, a total of 17 invalid questionnaires (missing questionnaires and blank questions) are eliminated, 148 valid questionnaires are obtained, and the effective questionnaire recovery rate is 89.7%. SPSS25.0 software (SPSS Inc., Chicago) is used to analyze the data collected.

## Results and Discussion

### Evaluation Results of Enterprise Talent Demand Model

**Figure 4:**
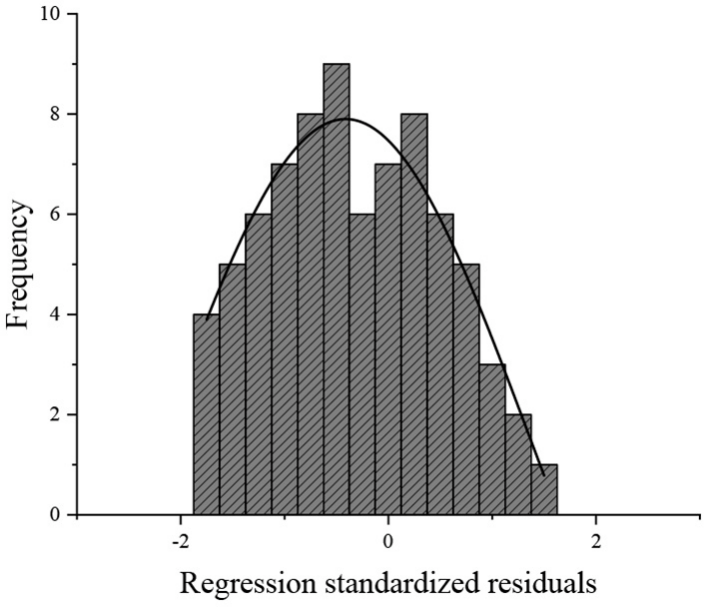
Histogram of normal curve.

**Figure 5:**
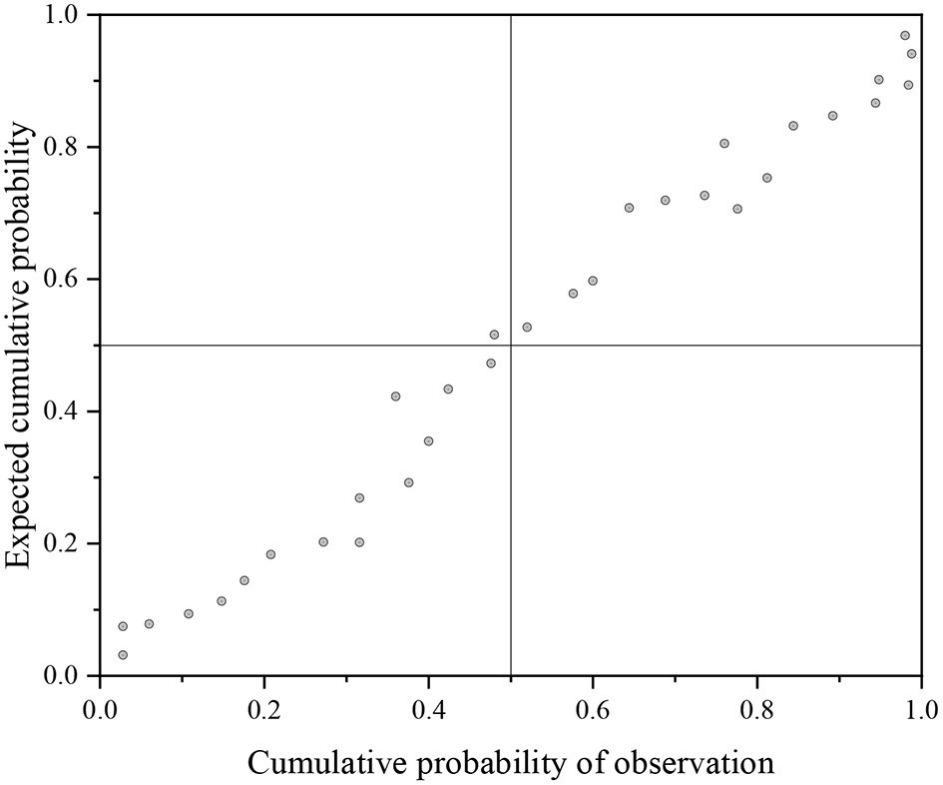
Standard p–p diagram.

The three steps of time series modeling are as follows: model establishment, parameter estimation, and model verification. Model verification is as important as model establishment and parameter estimation in time series modeling. After parameter estimation, hypothesis testing is needed to test the adaptability of the sequence to the ARIMA model. SPSS software is used to test the normality of the sequence, and “standardized residual histogram” is used to judge the residual distribution. If the residuals are normally distributed, the histogram of the normal curve is obtained as shown in Figure 4, and the standard p–p diagram reflects the normal distribution (Figure 5). The p–p diagram can directly detect the positive distribution of sample data by drawing the scatter of cumulative probability of variables corresponding to the cumulative probability of theoretical distribution.

In the histogram of the normal curve shown in Figure 4, the curve is bell-shaped distribution, which conforms to normal distribution. The research results are in line with the research expectations, so the follow-up analysis and prediction can be carried out. In the normal probability diagram shown in Figure 5, the data points are distributed near the straight line, and the extreme value is located on the straight line. Therefore, the error term of the ARIMA model cannot refuse the assumption of normality, and the time series model has high parameter setting accuracy.

### Analysis of the Prediction Results of Enterprise Talent Demand Model

In order to show the prediction results of the BP–ARIMA combination model more intuitively, it is necessary to introduce analysis indexes to evaluate the prediction results, to test the reliability and adaptability of the model. Root mean square error (RMSE) is used as the evaluation index of the performance of the enterprise demand prediction model. RMSE is defined as the sum of the squares of the error between the fitting value and the true value and the square root of the ratio of the number of observations *n*. The smaller the value is the higher the prediction accuracy of the model is.

(49) $$\begin{array}{l} RMSE=\sqrt{\frac{\overset{N}{\underset{I=1}{\sum }}{\left(X_{o,i}-X_{m,i}\right)}^{2}}{n}} \end{array}$$

where *X*_*o,i*_ is the original value, *X*_*m,i*_ is the predicted value, and *n* is the data length.

**Figure 6:**
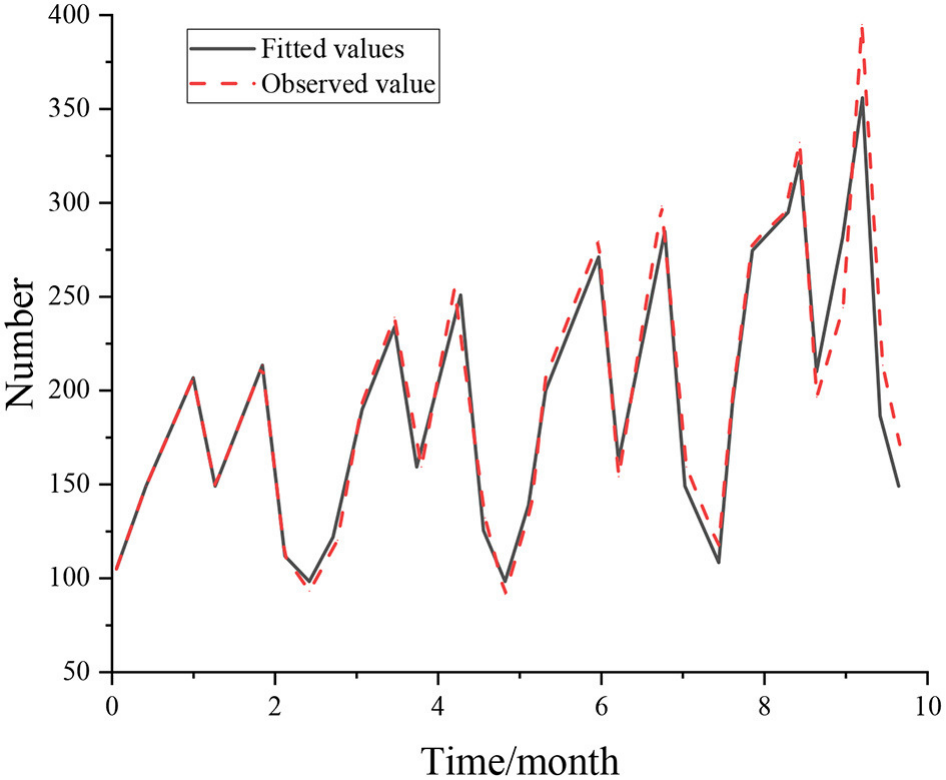
Trend forecast results.

The real value of demand and model fitting value of big data post is substituted into the equation, the RMSE index value is 6.374, and the model error is small, that is, the model has a better prediction effect and achieves the expected effect. The BP–ARIMA network model is used to forecast the demand in the next year, and the trend chart of the output of the predicted value is obtained (Figure 6).

Figure 6 shows that the results are consistent with those of the previous model and has good fitting effect. The potential demand of enterprises for big data talents in universities in the future can be obtained by fitting the prediction sequence. Based on the BP–ARIMA combination model, post modeling and prediction can provide data support for the research on the change and trend of talent demand in the field and provide a decision-making basis for a higher matching degree of talent cultivation.

### Analysis on the Matching Between Enterprise Talent Demand and College Talent

**Figure 7:**
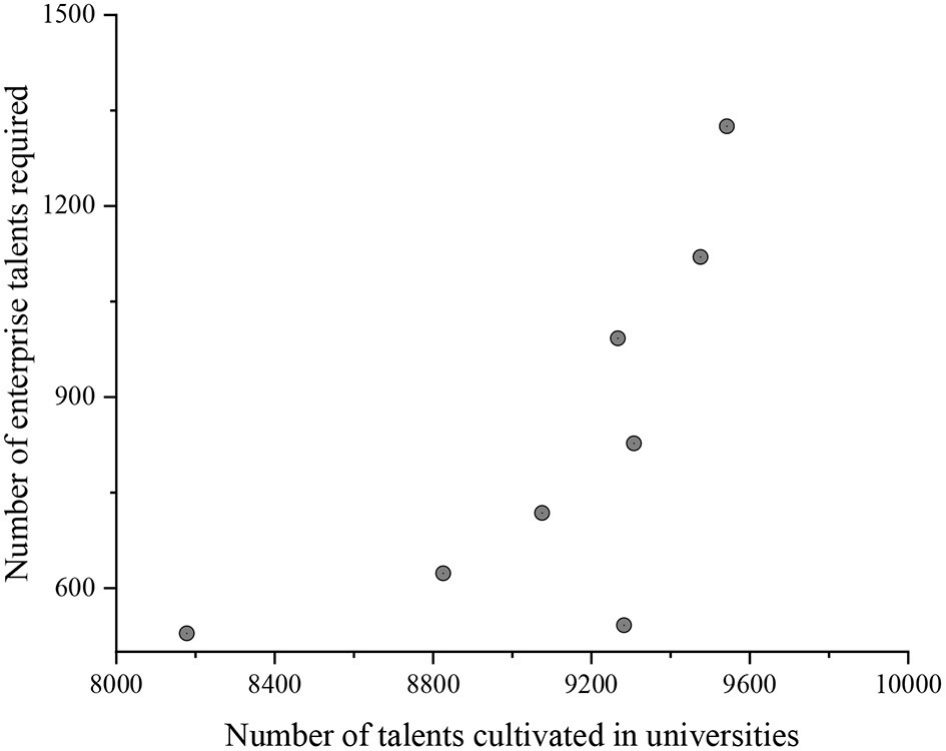
Scattered chart of the number of talents cultivated in universities and the number of talent demand of enterprises.

The BP–ARIMA combination model is used to predict the talent demand of big data-related enterprises in the next 4 years through recursive prediction. SPSS software is used to draw the scatter diagram of college specialty settings and enterprise demand (Figure 7).

Figure 7 reflects that the scatter chart of college specialty setting and enterprise demand tends to be a straight line, and the sequence has an obvious correlation. At present, the relationship between the two is further quantified by using the Pearson's correlation coefficient method. The results of the correlation coefficient are between 0 and 1. The results show that the Pearson's correlation coefficient is 0.6235. There is a positive correlation between the observed values *X* and *Y*, and the correlation degree between them is medium, that is, the matching degree between the cultivation of innovative talents in universities and the trend of demand for big data-related jobs needs to be strengthened. In order to meet the needs of big data-related posts in the future and further output talents in line with the enterprise, the professional cultivation program can be appropriately optimized.

### Analysis Results of the Mental Health of Young Entrepreneurs

**Figure 8:**
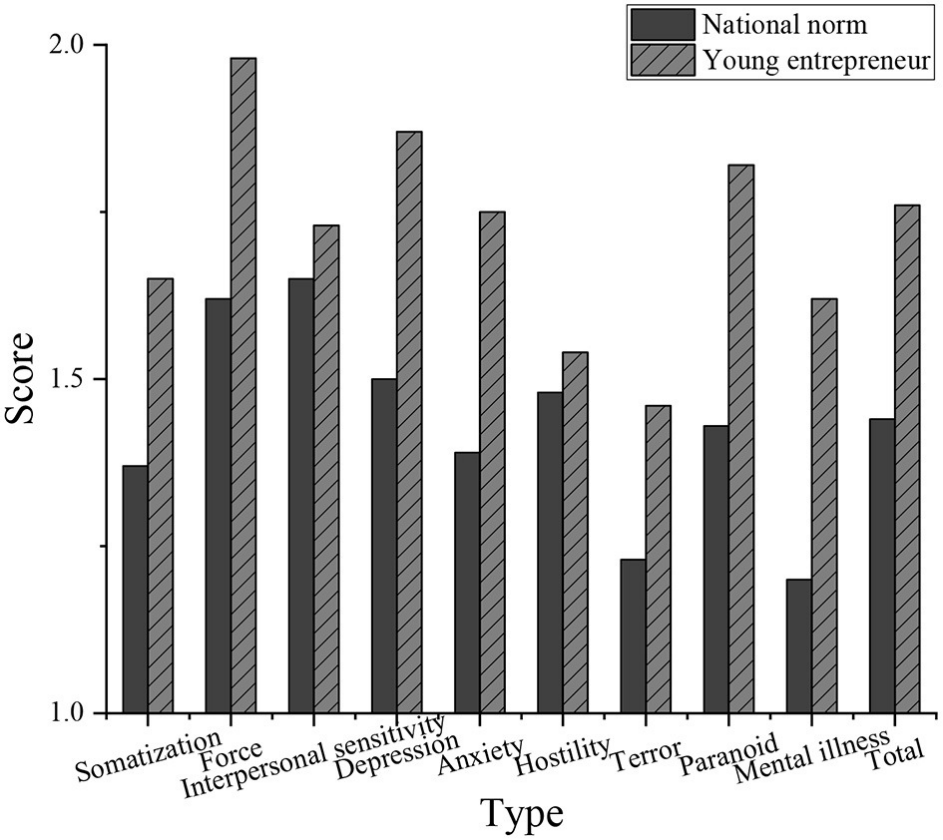
Comparison between the mental health level of young entrepreneurs and the national norm.

The SCL-90 scale is used to investigate the work and life status of young entrepreneurs and the overall level of mental health. Figure 8 shows the result of the data analysis.

The national norm is *N* = 1060. The total average score and factor scores of the SCL-90 scale of mental health of the new generation private entrepreneurs are significantly higher than that of the national norm (*p* < 0.01). It indicates that the mental health level of the new generation of private entrepreneurs is lower than that of the national norm (Tian et al., 2020).

**Figure 9:**
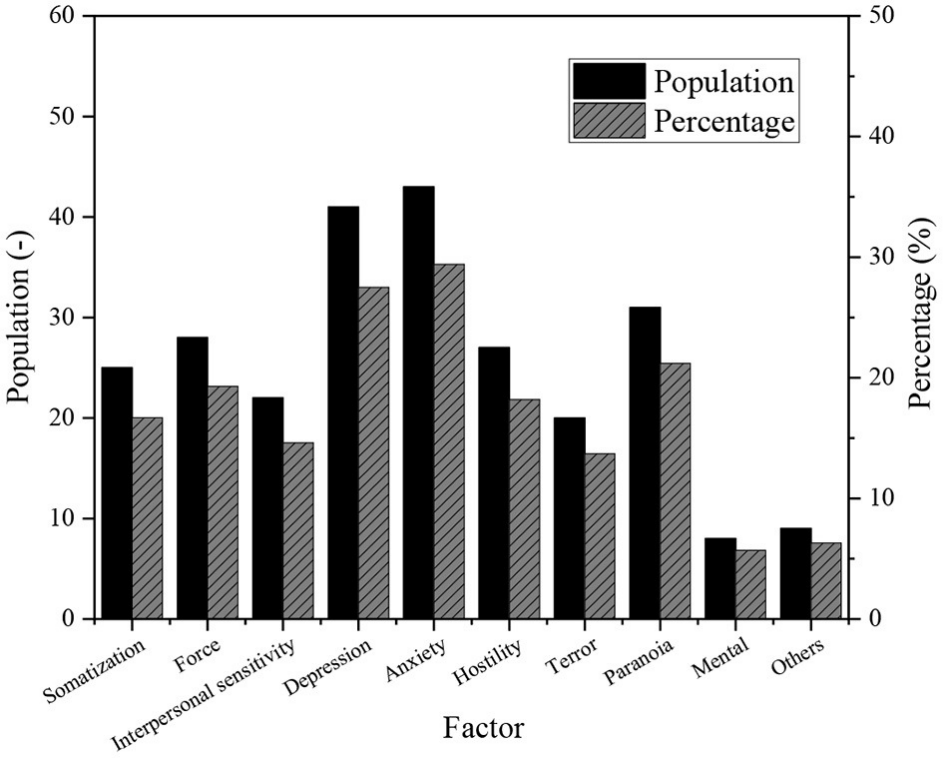
Number of SCL-90 positive young entrepreneurs.

Any factor score of SCL-90 > 2 is used as a positive detection index of mental health problems. According to this index, the number of positive people is 84 in this survey, and the positive detection rate is 56.7%. Figure 9 shows the number and proportion of SCL-90 positive people.

The results of SCL-90 show that the positive rate of mental health symptoms is 56.7% in the survey of 168 young private entrepreneurs in the effective sample. The subjects with positive depression and anxiety account for 27.5% and 29.4% in total, respectively. It suggests that there is a high level of depression and anxiety in young entrepreneurs. Compared with the national norm, the mental health level of young entrepreneurs is significantly lower than the national norm, and the mental health problems are generally serious.

In view of the above situation, the mental health of young entrepreneurs needs to be focused on promoting the healthy development of the mental health of young entrepreneurs.

## Conclusion

The matching analysis based on Pearson's correlation coefficient is carried out between it and the corresponding professional settings in universities. The conclusion is that on the whole, the mental health level of young entrepreneurs is lower than the national norm, and there is a high proportion of depression and anxiety. Considering the professional characteristics and competitive environment of young entrepreneurs, busy work and multiple missions given by society become the main sources of pressure. Therefore, in higher education, it is necessary to emphasize and strengthen the cultivation of psychological quality and mental health of talents, actively guide innovative talents to understand their own mental health, and pay attention to prevent the occurrence of mental health problems. However, due to the limitations, there are few young entrepreneurs studied and no long-term follow-up investigation on the objects. Hence, the follow-up study will further deepen the scope, establish a long-term survey of the psychological condition of young entrepreneurs, and provide more sufficient information for the related research of the psychological health of entrepreneurs.

## Data Availability Statement

The raw data supporting the conclusions of this article will be made available by the authors, without undue reservation.

## Ethics Statement

The studies involving human participants were reviewed and approved by Ningbo University Ethics Committee. The patients/participants provided their written informed consent to participate in this study. Written informed consent was obtained from the individual(s) for the publication of any potentially identifiable images or data included in this article.

## Author Contributions

All authors listed have made a substantial, direct and intellectual contribution to the work, and approved it for publication.

## Conflict of Interest

The authors declare that the research was conducted in the absence of any commercial or financial relationships that could be construed as a potential conflict of interest.

## Publisher's Note

All claims expressed in this article are solely those of the authors and do not necessarily represent those of their affiliated organizations, or those of the publisher, the editors and the reviewers. Any product that may be evaluated in this article, or claim that may be made by its manufacturer, is not guaranteed or endorsed by the publisher.
